# Society of Homœopathicians—A Reply

**Published:** 1895-03

**Authors:** 


### SOCIETY OF HOMŒOPATHICIANS.

### A REPLY.

TO THE EDITOR OF THE HOMŒOPATHIC PHYSICIAN:—In reply to the various criticisms of the preamble of the Society of Homœopathicians, which appeared in the January number of THE HOMŒOPATHIC PHYSICIAN, we have this to say:

The Executive Committee of the Society of Homœopathicians are fully cognizant of all the particulars leading up to the action of the I. H. A. at Narragansett Pier, June 21st, 1892, which resulted in the suppression of the report of the Board of Censors.

The preamble as published in the November number of THE HOMŒOPATHIC PHYSICIAN is, from beginning to end, *a statement of facts.* Consequently there is nothing to qualify or retract.

J. A. BIEGLER,
EDMUND CARLETON,
SAMUEL A. KIMBALL,
EUGENE W. SAWYER,
RUFUS L. THURSTON,
*Executive Committee.*

---

